# The extra-articular impacts of rheumatoid arthritis: moving towards holistic care

**DOI:** 10.1186/s41927-018-0039-2

**Published:** 2018-10-30

**Authors:** I. C. Scott, A. Machin, C. D. Mallen, S. L. Hider

**Affiliations:** 1 Research Institute for Primary Care & Health Sciences, Primary Care Sciences, Keele University, Newcastle-under-Lyme, Staffordshire UK; 2 Department of Rheumatology, Haywood Hospital, High Lane, Burslem, Staffordshire UK

**Keywords:** Rheumatoid arthritis, Pain, Fatigue, Mental health, Cachexia

## Abstract

Although treat-to-target has revolutionised the outcomes of patients with rheumatoid arthritis (RA) there is emerging evidence that attaining the target of remission is insufficient to normalise patients’ quality of life, and ameliorate the extra-articular impacts of RA. RA has a broad range of effects on patient’s lives, with four key “extra-articular” impacts being pain, depression and anxiety, fatigue and rheumatoid cachexia. All of these are seen frequently; for example, studies have reported that 1 in 4 patients with RA have high-levels of fatigue. Commonly used drug treatments (including simple analgesics, non-steroidal anti-inflammatory drugs and anti-depressants) have, at most, only modest benefits and often cause adverse events. Psychological strategies and dynamic and aerobic exercise all reduce issues like pain and fatigue, although their effects are also only modest. The aetiologies of these extra-articular impacts are multifactorial, but share overlapping components. Consequently, patients are likely to benefit from management strategies that extend beyond the assessment and treatment of synovitis, and incorporate more broad-based, or “holistic”, assessments of the extra-articular impacts of RA and their management, including non-pharmacological approaches. Innovative digital technologies (including tablet and smartphone “apps” that directly interface with hospital systems) are increasingly available that can directly capture patient-reported outcomes during and between clinic visits, and include them within electronic patient records. These are likely to play an important future role in delivering such approaches.

## Background

The current treatment paradigm for patients with rheumatoid arthritis (RA) is “treat-to-target” (T2T) [1]. This involves measuring a patient’s disease activity, using composite scores like the disease activity score on a 28-joint count (DAS28), and escalating disease-modifying anti-rheumatic drug (DMARD) therapy until the targets of remission, or low disease activity (LDA) are attained. The T2T strategy is based on the extensive evidence that patients attaining remission have better health related quality of life (HRQoL) and function, and lower rates of radiological damage, when compared to patients in higher disease activity states [2–6].

**Fig. 1:**
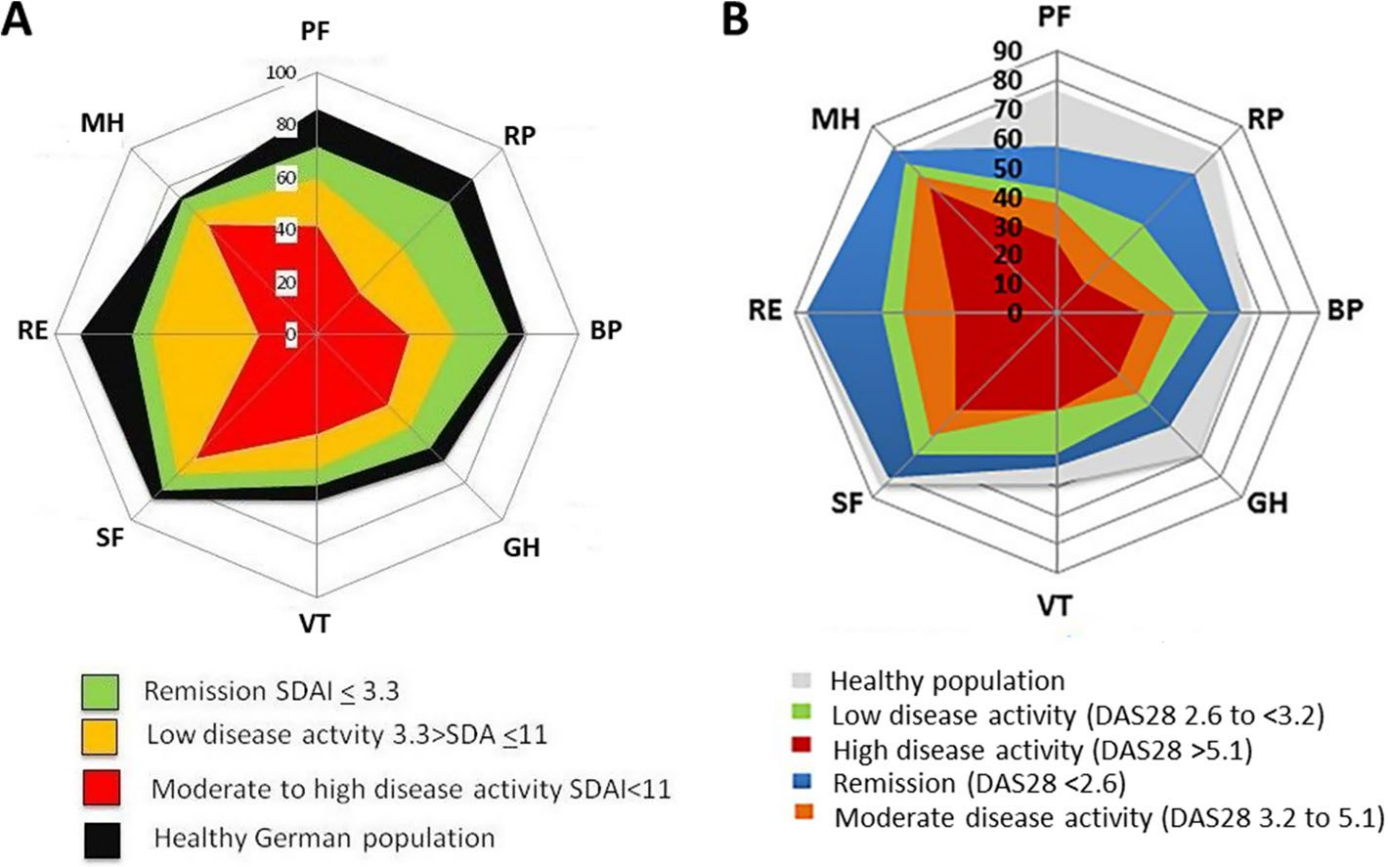
Spydergrams Showing Impact of Attaining Remission on Short-Form 36 Health Profiles in Patients with Established RA. Panel A = SF-36 health profiles in German RA patients, stratified by disease activity status (captured using the simplified disease activity index) and compared to the healthy German population. Panel B = SF-36 health profiles in 205 English RA patients enrolled to the TACIT trial at the end-point of 12-months, stratified by disease activity status (captured using the DAS28). PF = physical functioning, RP = role physical, BP = bodily pain; GH = general health; VT = vitality; SF = social functioning, RE = role emotional; MH = mental health. Figures adapted with permission under the creative commons attribution license from the original published papers [5, 7]

RA has many impacts on patients’ lives not directly addressed by reducing disease activity using T2T strategies. Four key examples are [1] pain, [2] depression and anxiety, [3] fatigue, and [4] muscle loss. Although controlling disease activity and achieving remission benefits patients it usually fails to normalise HRQoL [5, 7] and ameliorate pain [8] and fatigue [9, 10]. This is particularly true of those individuals with established disease, with two independent studies showing that short-form 36 (SF-36) health profiles – measuring health across 8 domains, each of which is scored from 0 to 100, with higher scores representing better health – are worse in patients with established RA in remission, compared with the normal general population (Fig. 1) [5, 7]. The first study by Radner et al. [5], compared SF-36 health profiles in 356 German RA patients at a single time-point stratified by disease activity status (captured using the simplified disease activity index) to those observed in the healthy German population; lower HRQoL was seen in all 8 domains in patients in remission compared with the healthy population. The second study, by Scott et al. [7], compared SF-36 health profiles in 205 English RA patients enrolled to the TACIT trial (of combination DMARDs vs. anti-TNF) at the trial end-point of 12-months, stratified by disease activity status (captured using the DAS28); lower HRQoL was seen in all domains in patients in remission, with the exception of mental health. The impact of RA on HRQoL is likely to be minimised by extending the focus of disease management beyond synovitis, to incorporate the evaluation of issues like pain, depression and anxiety.

In this review we will provide an overview of pain, depression and anxiety, fatigue, and muscle loss in patients with RA. We have focussed on these four “extra-articular” impacts as they are a diverse group of features, which have been studied in detail, are relatively common, improve with readily available interventions, have negative impacts on patients’ lives including reducing their HRQoL, and cannot be resolved simply by achieving remission. We will summarise their prevalence, aetiology, assessment tools, and treatment strategies. We will also outline the ways in which they can be assessed within routine practice settings.

### Pain

#### Definition

The conventional definition of pain from the International Association for the Study of Pain. defines it as “an unpleasant sensory and emotional experience associated with actual or potential tissue damage, or described in terms of such damage [11]”. This broad definition reflects the multidimensional nature of pain, which is purely subjective, harbours an emotional element, and can occur in the absence of actual tissue damage. At the same time, it is important to appreciate that there are divergent views on how to define pain. For example, McCaffery defined pain to be “whatever the experiencing person says it is, existing whenever the experiencing person says it does” [12].

#### Assessing pain

A broad range of patient reported outcome (PRO) instruments have been developed and used to capture pain in patients with RA. Burkhardt and Jones have published a detailed summary of their assessment of the key measures [13]. An overview of these is provided in Table 1. They span quick and simple unidimensional instruments of pain intensity such as the pain VAS [14], generic multidimensional instruments such as the McGill Pain Questionnaire [15, 16] (capturing information on many pain dimensions across a range of adult populations), and disease-specific instruments like the RA pain scale (RAPS) [17] (gaining information on pain most relevant to patients with RA).

**Table 1:** Key Methods to Assess Pain in Patients with Rheumatoid Arthritis

Measure	Population	Content	Completion time (minutes)	Scoring time (minutes)
McGill Pain Questionnaire [15, 16]	For use in adults with chronic pain problems	78 words describing the sensory, affective and evaluative aspects of pain, alongside a 5-point present pain intensity scale.	5–15	1–2
Rheumatoid Arthritis Pain Scale [17]	Adults with RA	24 items measuring descriptions of pain, it’s severity and interference.	5	2
Pain Visual Analogue Scale [14]	Any adult population	Usually one horizontal line, measuring 10 cm, anchored with verbal descriptors “no pain” and “pain as bad as it could be”.	< 1	< 1
Verbal Descriptive Scale [14]	Any adult population	Similar to pain visual analogue scale, replacing whole numbers with verbal descriptors of pain (e.g. no pain, slight pain, mild pain, moderate pain, severe pain, very severe pain, the most intense pain imaginable).	< 1	< 1
Numeric rating scale [123]	Any adult population	Segmented version of pain visual analogue scale, with patients selecting a whole number (0–10 integers) that best reflects their pain intensity	< 1	< 1
Short-Form 36 Bodily pain [124]	Any adult population	A 2-item scale in which patients rate: [1] the intensity of their pain (6-point scale ranging from “none” to “very severe”), and [2] extent to which pain interferes with their work (5-point scale ranging from “not at all” to “extremely”)	< 2	1

The simplest to use within a busy, routine clinical setting is the pain VAS [14]. This comprises one horizontal or vertical line, commonly 10 cm long, that has the verbal descriptors “no pain” and “pain as bad as it could be” at either end (although variations in verbal end-points are often observed). Patients place a line perpendicular to the VAS line at the point best representing their current pain, with the score ranging from 0 to 100 (if scored in mm). The pain VAS has been shown to have high test-retest reliability in patients with RA, although it is higher in literate (*r* = 0.94) than illiterate (*r* = 0.71) people [18]. The optimal cut-off to define an “acceptable” level of pain has been defined as ≤2.0 units, and the minimal clinically important change for pain in observational studies reported as being 1.1 units [19]. Whilst the pain VAS is easy to score and interpret, as it is a unidimensional measure it cannot fully capture the multidimensional nature of patients’ pain.

#### Prevalence in RA

Pain represents a key symptom in patients with RA. In the earliest stages of the disease process, it is the dominant reason why people initially seek a review by their physician, with a recent qualitative study of patients with newly diagnosed RA reporting pain to be central to their symptom experience [20].

In patients with established RA, pain is also an important issue. In two multi-national RA patient surveys – the “Good Days Fast” survey, which explored the impact of RA on the lives of women, and the “Getting to Your Destination Faster” survey, which explored patients’ treatment goals – pain was identified as being of paramount importance [21]. In the “Good Days Fast” survey, from 1958 women surveyed, 63% reported experiencing pain every day, with 75% taking analgesics. Despite the high prevalence of pain, however, many patients reported problems discussing it with their health care provider, with 55% feeling too shy to talk about how much pain they experienced, and 73% reporting they feel like they are complaining when discussing their pain symptoms. In the “Getting to Your Destination Faster” survey, from the 1829 patients surveyed, 70% agreed that pain relief was the most important aspect of their management. A further third survey, of 1024 patients with RA in Norway, showed similar findings. In this study, 69% of patients reported pain as their preferred area for improvement [22], despite which over one-third of patients were not receiving analgesics. Taken together, these three patient surveys provide good evidence that improving pain is a crucial, patient-centred treatment goal in RA.

#### Aetiology of RA pain

Pain in patients with RA is multifactorial. Synovitis, systemic inflammation [23], and joint damage [24] all play roles in both the initiation and perpetuation of pain. However, pain also often occurs in the absence of synovitis or joint damage, highlighting the importance of peripheral sensitisation (hypersensitivity of the nociceptive primary afferent neurons in the peripheral nervous system) and central sensitisation (hyperexcitability of nociceptive neurons in the central nervous system) [25].

High levels of pain are generally observed in patients with highly active disease, and improve with the use of intensive synthetic and biologic DMARD therapy [26]. Although reducing synovitis with intensive DMARD treatment improves pain, in many patients clinically significant levels of pain remain in the absence of synovitis. This is demonstrated in an analysis of the North American Brigham and Women’s Hospital RA Sequential study (BRASS), by Lee et al. [8]. In this analysis, the 154 patients in DAS28-CRP defined sustained remission over 12 months were evaluated; 11.9% had clinically significant pain at baseline (defined as a multi-dimensional health assessment (MDHAQ) pain score of ≥4) and 12.5% after 1 year of follow-up. Pain scores were observed to be significantly and positively associated with fatigue and sleep disturbance (evaluated using the MDHAQ), and significantly and negatively associated with self-efficacy (evaluated using the arthritis self-efficacy score). No significant association with inflammatory markers or seropositivity was reported. Other studies have also reported pain scores above those seen in the normal population in patients with RA in remission [5, 7].

There is strong clinical and experimental evidence that peripheral and central sensitisation play crucial roles in RA-related pain. This has led to the use of the term “fibromyalgic RA”, in which fibromyalgia and RA co-exist in the same patient [27]. The prevalence of co-existing fibromyalgia in people with RA is high; a large study of 11,866 patients with RA identified 1731 (17.1%) as also having fibromyalgia, the presence of which associated with increased medical costs, more severe RA, and a worse HRQoL [28]. Animal studies provide further evidence for the role of pain pathway aberrancies in inflammatory arthritis, with these seeming to occur prior to the onset of clinical signs of synovitis. Nieto et al. evaluated this issue in two separate studies of female rodents with a collagen-induced arthritis. In the first study, allodynia of the rodent hind paw developed concomitantly with articular inflammatory cell infiltration, activation of joint nociceptors, and spinal microgliosis; these changes took place prior to the onset of visible synovitis. When paw swelling finally developed, a significant number of primary afferent neurons innervating tissues external to the joint were also activated [29]. In the second study, they reported that mechanical allodynia was evident prior to the development of visible paw swelling, worsened as swelling developed, and was associated with reactive spinal microgliosis [30]. Microglial cells are resident macrophages in the central nervous system [31], which rapidly respond to a broad range of stimuli. They appear critical to the development of chronic pain and central sensitisation [32], with activated microglia secreting pro-inflammatory and pro-nociceptive mediators, such as TNF and IL-18, which modulate synaptic transmission and pain [33, 34].

Whilst it is often perceived that joint damage is a contributor to pain, the evidence for this is, at best, limited. Sokka et al. evaluated the relationship between Larsen scores and function (assessed using the health assessment questionnaire (HAQ)) and pain (assessed using the pain visual analogue scale (VAS)) in 141 patients with established RA [35]. Larsen scores had a signification association with HAQ (*r* = 0.277, *P* = 0.001) but not pain VAS (*r* = 0.008, *P* = 0.929). Sarzi-Puttini et al. also evaluated associations between cross-sectional pain VAS, and disease characteristics and outcomes in 105 patients with established RA [24]. In a multivariate regression model, Larsen scores explained only 2.1% of the variation in pain VAS.

#### Treatment of pain in RA

The multifactorial and multidimensional nature of pain suggests that a multifaceted approach to its management is needed that combines pharmacological strategies, with psychological and physical therapies, which have been demonstrated across a range of trials to have beneficial effects on reducing RA pain.

DMARDs and biologics reduce pain in active RA, and optimising immunosuppressive therapy to control RA is important in this regard. In addition, both simple analgesics such as paracetamol and non-steroid anti-inflammatory drugs (NSAIDs) also reduce pain levels, although their effects are generally small-to-modest. Hazelwood et al. systematically reviewed the evidence for the efficacy of paracetamol in inflammatory arthritis, identifying 12 trials and 1 observational study [36]. There was weak evidence of a benefit of paracetamol over placebo. However, most of the included studies were reported 20–50 years ago, and some evaluated atypical paracetamol dosing (such as 2 g of paracetamol over 24-h [37]). Additionally, they had high-risks of bias due to incomplete reporting of details surrounding sequence generation, allocation concealment, and blinding, alongside incomplete outcome data with high dropout rates and lack of intention-to-treat analysis. NSAIDs are commonly used in patients with RA, with clinical trials supporting their efficacy [38, 39]. Whilst clinicians and patients prefer to use NSAIDs over paracetamol in RA, the relative analgesic merits of NSAIDs compared with paracetamol are uncertain [40].

Opiates are prescribed to a substantial minority of patients with RA. One observational study from North America found over one-third of RA patients used opiates in some form [41]. In more than a tenth use was chronic, with opiate use increasing in recent years. However, there is limited evidence for their efficacy. Whittle et al. systematically reviewed the literature for trials comparing opiates vs. another intervention or placebo in patients with RA. Eleven studies were identified, all of which were of a short duration (< 6 weeks). Although opiates were more likely to improve the patient-reported global impression of change in pain, they were also more likely to cause adverse events, with no difference in net efficacy after adjustment for adverse events observed between opioids and placebo [42].

Tricyclic anti-depressants and neuromodulators (such as nefopam) are also often used, particularly if patients have poor sleep or fibromyalgic RA. As with opiates, the evidence supporting their efficacy is weak, with systematic reviews reporting limited evidence that oral nefopam and topical capsaicin are superior to placebo at reducing pain in patients with RA [43], and inconclusive evidence about the efficacy of tricyclic antidepressants [44].

When these limited benefits are weighed against the toxicity profiles of these analgesics – with both paracetamol and NSAIDs associating with an increased risk of myocardial infarction, renal impairment, and upper GI bleeds [45–47], and nefopam and tricyclic antidepressants frequently causing side-effects – it appears vital to ensure that patients are fully informed of the risks and benefits of their analgesic treatment, and that they are used cautiously, for the shortest duration possible, and stopped if patients are failing to gain clinical benefit.

Exercise is encouraged in patients with RA, due to its wide-ranging impacts on general health and well-being. Exercise is defined as any activity that improves physical fitness. It can vary in type and intensity. Several trials have evaluated the impact of dynamic exercise (defined as activities with sufficient intensity, duration, and frequency to improve stamina or muscle strength) on pain in RA [48]. A systematic review reported small benefits on pain scores in patients receiving short-term, land-based aerobic capacity and muscle strength training, with patients receiving dynamic exercise rating their pain to be 0.5 units lower (on a 0–10 scale) at 12-weeks, compared to those not receiving the intervention [48]. However, this change is below the minimal clinically important difference for pain [49].

Psychological interventions are also a vital component of managing chronic musculoskeletal pain. These focus on empowering patients to self-manage their pain. Three commonly employed psychological strategies comprise: [1] stress management training, which helps patients cope with functional problems resulting from RA; [2] education, helping patients make informed decisions about self-managing their condition; and [3] cognitive-behavioural therapy (CBT), which teaches patients methods to manage their pain. Knittle et al. evaluated the effects of such face-to-face psychological interventions by undertaking a systematic review and meta-analysis of relevant randomised controlled trials. Small, but statistically significant effects were seen on improving physical activity, pain, disability and depression at follow-up evaluations [50]. Similar findings were reported in another systematic review of psychological interventions in RA, undertaken by Astin et al [51]; it found significant but small pooled effect sizes post-intervention for pain of 0.22.

### Anxiety and depression

#### Definition

Anxiety disorders are defined by excess worry, hyperarousal and fear which is both counterproductive and debilitating [52]. Its most extreme form is generalised anxiety disorder (GAD), which is characterised by persistently heightened tension and excessive worry about a range of events, that contributes to impaired functioning [53]. Depression is characterised by a persistently low mood, and loss of interest or pleasure in most activities. Depression may be associated with symptoms including an altered appetite, poor sleep, fatigue, lack of concentration and suicidal thoughts. The degree of depression is determined by the number and severity of associated symptoms, and any related functional impairment [54].

#### Prevalence in RA

Approximately 38% of patients with RA suffer from depression [55]. The prevalence of anxiety is approximately half that of depression, and estimated to lie between 13 and 20% [56, 57]. When this is compared to the prevalence of depression and anxiety in the general population (with the 2014 Adult Psychiatric Morbidity Survey reporting that 5.9% and 3.3% of the adult English population suffered from generalised anxiety disorder and a depressive disorder, respectively) [58], it is clear that patients with RA have a significantly increased mental health burden.

#### Aetiology in RA

Margaretten et al. have previously provided a summary of the multifactorial nature of reduced mental health in RA [59]. It is likely that different factors contribute to the initiation and perpetuation of depression in different individuals. Characteristics that have been associated with depression include low socioeconomic status [60], co-morbidities [61, 62], pain [23], and disability [63, 64]. Systemic inflammation has also been linked with depression, leading to the proposal of the “cytokine hypothesis of depression”, in which pro-inflammatory cytokines are considered to be important mediators of this disorder [65]. It remains to be determined, however, as to whether such cytokines are causally involved in depression aetiology, or if they represent immunological reactions to depressive disorders [65]. Additionally, in the context of RA, the link between systemic inflammation and the onset of depression is uncertain [23, 66].

The factors underlying the excess anxiety observed in RA have received less attention than those of depression. However, a recent review by Sturgeon et al. highlighted the key issues [67]. Anxiety in RA is driven in part by personal factors including social context combined with the impact of ongoing pain and disability and the inflammatory process. The factors causing depression and anxiety in RA are very similar and often occur together in individual patients.

#### Impacts

Comorbid mental health problems in RA are associated with worse patient outcomes. Several studies have reported that poorer mental health associates with higher levels of DAS28-defined disease activity, although this appears to be driven by its relationship with the “subjective” components of the DAS28 (the tender joint count (TJC) and patient global assessment of disease activity (PtGA)). Matcham et al.*.* performed a secondary analysis of the CARDERA trial, reporting that the presence of persistent depression and anxiety associated with higher DAS28 scores over time; exploring relationships with the individual DAS28-components revealed the association was restricted to the TJC and PtGA, with no significant association seen between depression and anxiety and the swollen joint count (SJC) and erythrocyte sedimentation rate (ESR) [68]. Similarly, Cordingley et al. reported a significant association between the PtGA and the Hospital Anxiety and Depression Scale (HADS) depression score in 322 RA patients awaiting biologic therapy, but not the other DAS28 components [69].

Depression has also been linked with increased mortality in RA, with Ang et al.*.* reporting that amongst 1290 patients with RA observed over 18 years, the presence of clinical depression in the first 4 years of entry into their clinical cohort provided a hazards ratio (HR) on mortality of 2.2 (95% CI 1.2–3.9, *P* = 0.01) [70]. Depression also increases healthcare costs, with Michaud et al. identifying the presence of depression to be a key predictor of increased medical outpatient costs (outpatient procedures, laboratory tests, and physician visits) amongst 7527 RA patients, followed up over a 2-year period [71].

#### Identifying anxiety and depression

Despite the detrimental impact of mental health disorders on RA outcomes, rheumatologists and primary care physicians do not routinely screen for the presence of mental health issues in patients with RA. In the National Health Service (NHS) this probably reflects a combination of time constraints within clinic appointments, alongside uncertainties as to who is leading on this aspect of patient care (primary or secondary care clinicians). However, to improve the outcomes and HRQoL of patients, the recognition and management of mood problems in RA should be a healthcare priority. Research from the Institute of Psychiatry in London has both highlighted the relative absence of screening in standard care for long-term conditions and shown it can be readily achieved using simple digital assessment methods [72].

One method to implement the routine screening of mental health disorders in RA would be to incorporate it within an annual review. This process is recommended by the National Institute for Health and Care Excellence (NICE), which advise an RA annual review that incorporates an assessment of mood. There are, however, several problems implementing this recommendation. Firstly, there is uncertainty as to where the annual review should occur, and although the NHS Quality and Outcomes Framework (QOF) – which focusses on improving the care of long-term diseases through financial incentives to attain specific clinical targets [73] – incentivises a primary-care based annual review of patients with RA, 20% of GPs feel that this does not benefit their patients [74]. Secondly, it is unclear how mental health should be assessed within an annual review. Thirdly, there is a lack of a standardised approach to the annual review process, with cardiovascular and osteoporosis risk assessments being undertaken more often than depression screening [74].

NICE guidelines for the identification of depression in adults with chronic physical health problems [75], suggest the most sensitive tools for case-finding are the General Health Questionnaire (GHQ-28) and the two-stem questions of the Patient Health Questionnaire (PHQ-9) [75], with the latter often preferred due to their ease of use. These two-stem questions comprise: [1] during the last month, have you often been bothered by feeling down, depressed or hopeless? and [2] during the last month, have you often been bothered by having little interest or pleasure in doing things?

International guidelines for identifying anxiety and experience from the Institute of Psychiatry in London suggests a similar approach can be taken to find patients with significant anxiety [72, 76]. An abbreviated version of the GAD-7 scale, the GAD-2, has been recommended as a case-finding tool for anxiety. This asks two questions: [1] during the last month, have you often been bothered by feeling nervous, anxious or on edge? and [2] during the last month, have you often been bothered by not being able to stop or control worrying? It has a moderately high balance of sensitivity and specificity for detecting clinically relevant anxiety [77].

#### Managing anxiety and depression in RA

**Fig. 2:**
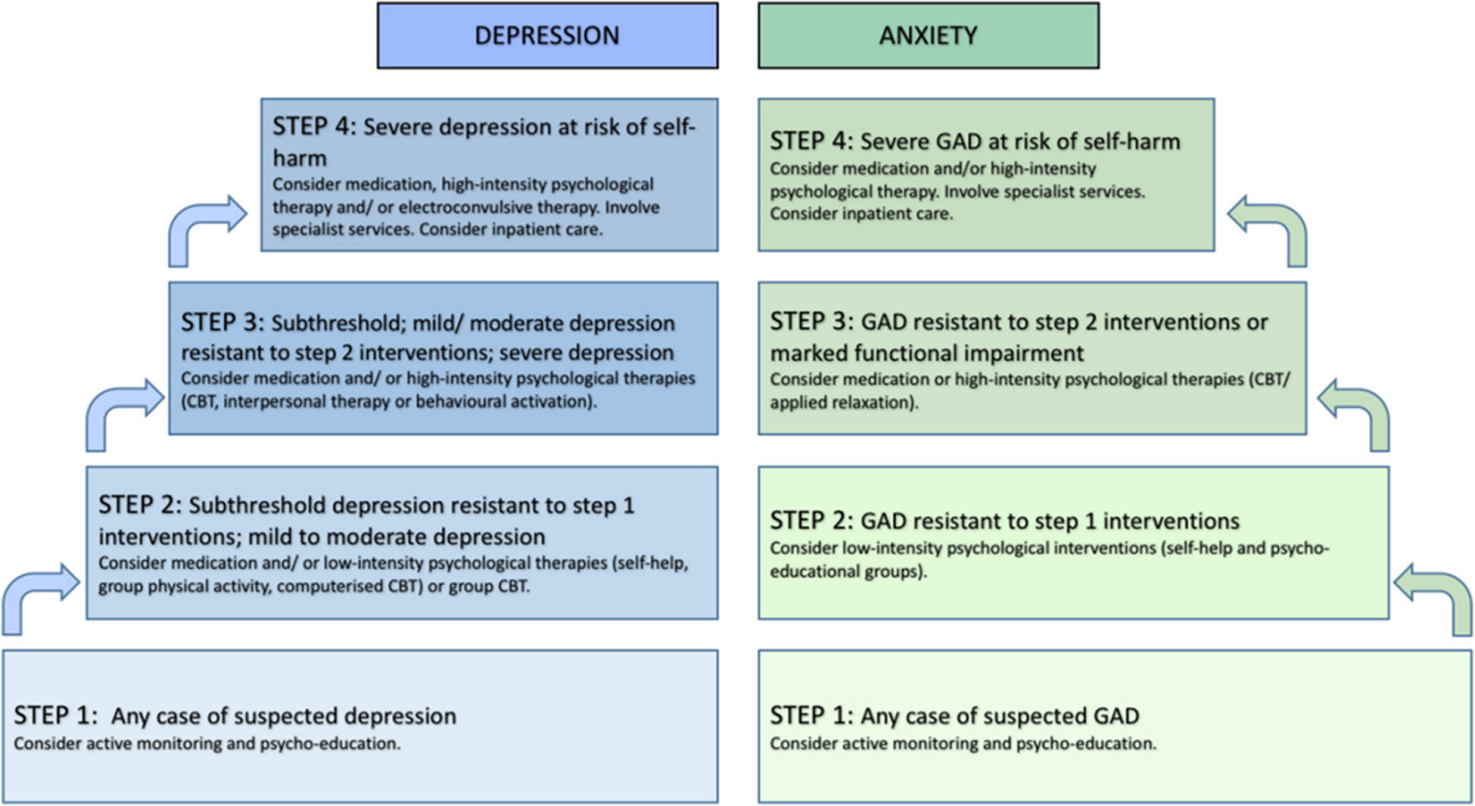
Stepped Care Approach to Managing Depression and Anxiety in Adults (based on NICE guidelines). CBT = cognitive behavioural therapy; GAD = generalised anxiety disorder. Figure produced using information provided in NICE guidelines for managing depression in adults [54] and adults with a chronic physical health problem [75], alongside guidelines for managing generalised anxiety disorder in adults [53]

NICE have produced guidelines for the management of depression and generalised anxiety disorder in adults, and also the management of depression in adults with long-term physical health disorders. These recommend a stepped care approach, outlined in Fig. 2, in order to identify the most effective, and least intrusive intervention [53, 54, 75]. If a person declines, or fails to benefit from a treatment, they are offered an appropriate intervention from the next step in the pathway.

Specific to patients with RA, only a handful of trials have evaluated interventions to treat depression and anxiety. A recently published systematic literature review has highlighted the paucity of data in this area [78]. This reviewed literature from controlled trials of treatments for depression and anxiety in RA. Only 8 trials were identified, all of which evaluated interventions for depression; no trials evaluated anxiety treatments. Of these, only one trial assessed medications that are often used in contemporary practice (comparing the selective serotonin reuptake inhibitor, paroxetine, with the tricyclic antidepressant, amitriptyline); the remainder used medications that are used infrequently, such as dothiepin and trimipramine, or Chinese herbal remedies. Only 1 trial evaluated non-pharmacological approaches alone, with another assessing a combination of drug and psychological interventions. Overall, a trend towards efficacy was observed with active pharmacological treatments (standardised mean difference − 0.49; 95% CI -1.07 to 0.10), although this was not significant, and significant heterogeneity was observed between study estimates. The one trial of a psychological intervention (randomising 30 patients to cognitive behavioural therapy, and 29 patients to usual care) reported no statistically significant effect on depressive symptoms [79]. Overall, the level of evidence identified by this review was only low-to-moderate, and further research is required before more definitive conclusions can be made regarding pharmacological and non-pharmacological interventions to manage depression and anxiety in RA.

#### Patient perspectives on management approaches

Qualitative research suggests that patients with RA and comorbid anxiety and depression would favour the use of psychological, over pharmacological interventions. Machin et al. interviewed patients with RA who responded positively to the case-finding questions for anxiety and/or depression (using GAD-2 and/or PHQ-2), to explore their perspectives on this issue [80]. This was conducted in one clinic in England. In the quantitative part of the study 171 patients attending a nurse-led annual review clinic completed the questionnaire; scores in 28% suggested they were anxious or depressed. Fourteen of the patients participated in the qualitative study. They were predominantly white women (68%) reflecting the ethnicity of the local population and the prevalence of RA in females; their average was 63 years and the majority were retired. Patients with mental health problems felt considerable shame and stigma mentioning them to their clinicians. Whereas some participants were open to pharmacological treatments, others feared potential drug interactions, or perceived that medication was offered as a “quick fix”. Overall, participants expressed a preference for psychological therapies, although several reported difficulties accessing such care.

This preference for psychological treatments was replicated in a study exploring 46 US Hispanic patients’ perspectives of depression associated with RA. Patients often perceived antidepressants to be unnecessary or associated with side-effects, with a preference expressed for interventions incorporating an interpersonal component, such as support groups [81]. A third study, which represented a survey of 2280 patients with inflammatory arthritis that focussed on exploring patient views on their psychological support, also identified a substantial demand for psychological interventions [82]. Of the 1210 respondents, approximately two-thirds reported that they would use a self-management/coping clinic if the service were offered.

Despite these patient preferences, rheumatology units within England self-report a lack of access to psychological support. A postal survey to rheumatology units in 143 acute trusts across England highlighted this issue. Of the respondents, 73% rated their unit’s psychological support provision as being “inadequate”, despite most feeling that psychological support fell within their remit [83]. Barriers to providing psychological support included clinical time constraints, a lack of available training, alongside delivery costs.

### Fatigue

#### Definition

Fatigue is defined as a state of exhaustion and decreased strength accompanied by a feeling of weariness, sleepiness, and irritability, with a cognitive component [84]. It is unrelated to energy expenditure, and does not improve with rest.

#### Prevalence in RA

Fatigue is an extremely common symptom in RA. In the Quantitative Standard monitoring of Patients with RA (QUEST-RA) study (evaluating 9874 patients, across 34 countries) high levels of fatigue (defined as a Fatigue VAS of > 6.6 units) were found in almost 1 in 4 patients [85]. A recent systematic review of RA-fatigue aetiology reported that amongst 121 studies (totalling > 100,000 patients with RA) the mean fatigue score (on a normalised scale ranging from 0 (no fatigue) to 1.0 (worst possible fatigue)) was 0.5 units [86].

#### Aetiology of fatigue in RA

**Fig. 3:**
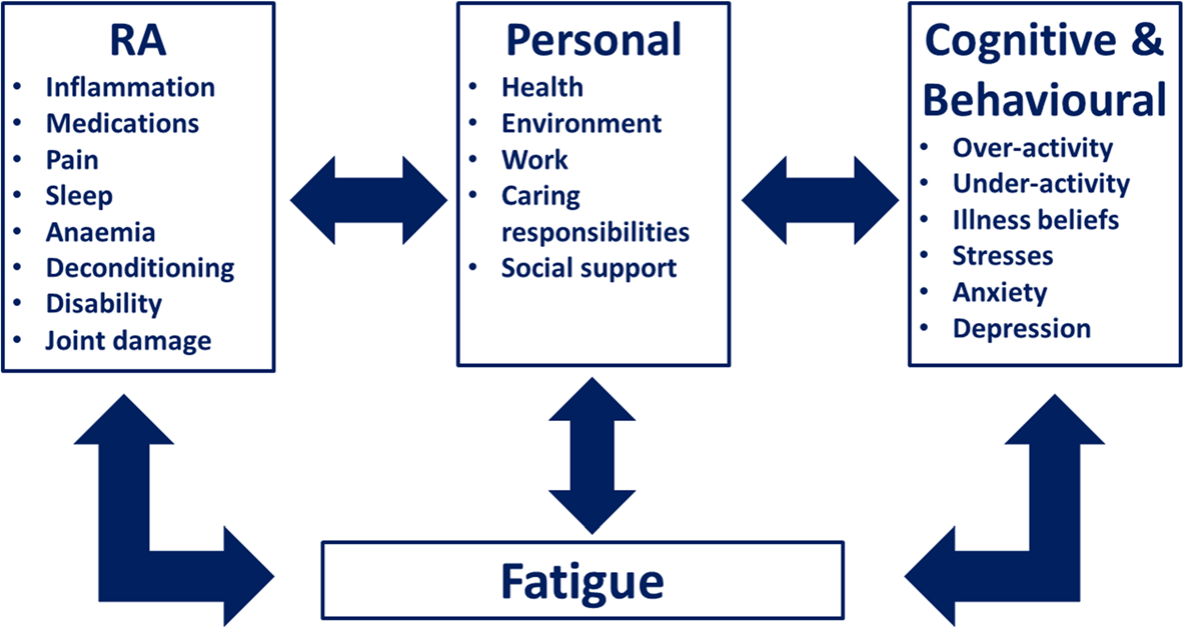
Conceptual Model for RA-Related Fatigue Proposed by Hewlett et al [87]. Figure produced using concepts reported by Hewlett et al [87]

The aetiology of fatigue in RA appears multifactorial. Hewlett et al. proposed a conceptual model for RA-related fatigue, to facilitate research into causal pathways and interventions. This conceptual model has three core, interacting components: [1] the RA disease process (RA), [2] thoughts, feelings and behaviours (cognitive, behavioural) and [3] personal life issues (personal) [87]. An overview of the key proposed factors in each of these components is provided in Fig. 3. This conceptual model highlights the substantial interaction that is considered to occur between fatigue, pain and disability.

Since the publication of this conceptual model, several systematic reviews have assessed factors associated with RA-fatigue. A recent systematic review of 121 studies, by Madsen et al.*.*, reported positive associations between fatigue and pain, CRP, ESR and DAS28. They also reported that high levels of fatigue occurred even in patients with well controlled disease [86]. An earlier systematic review of 25 studies by Nikolaus et al, reported that the relationship between fatigue and many variables is uncertain, with conflicting evidence observed across studies (particularly with regards to characteristics of inflammatory activity) [88]. However, the most convincing evidence for a relationship with fatigue was observed for pain, disability, and depression.

#### Assessing fatigue

There are multiple methods to measure fatigue in RA, which have previously been reviewed in detail by Hewlett and colleagues in two reviews [87, 89]. We have provided a summary of some key methods in Table 2. As with assessing pain, the quickest and simplest way to measure fatigue, and therefore the method that may be preferable to use in routine care, is using a VAS (scoring 0 to 100, with higher scores indicating greater fatigue). As with the pain VAS, as it is a unidimensional measure it cannot fully capture the multidimensional nature of patients’ fatigue.

**Table 2:** Key methods to assess fatigue in patients with rheumatoid arthritis

Measure	Population	Content	Completion time (minutes)	Scoring time (minutes)
Bristol RA Fatigue Multi-Dimensional Questionnaire (BRAF MDQ) [125]	Adults with RA	20 items cover domains of physical fatigue, living with fatigue, cognitive fatigue, and emotional fatigue.	5	3
Bristol RA Fatigue Numerical Rating Scales (BRAF NRS) For Severity, Effect, And Coping [125]	Adults with RA	3 single-item numeric rating scales on fatigue severity, effect on patients’ lives, and coping with fatigue.	1	1
Fatigue Visual Analogue Scale [89]	Any adult population	Usually one horizontal line, measuring 10 cm, anchored with verbal descriptors such as “not at all tired” and “very tired”.	< 1	< 1
Functional Assessment Chronic Illness Therapy (Fatigue) (FACIT-F) [126]	Adults with chronic illness	13 items covering physical fatigue, functional fatigue, emotional fatigue, and social consequences of fatigue.	4	4
Multi-Dimensional Assessment of Fatigue [127]	Adults with RA	15 items covering 4 dimensions of fatigue: severity, distress, interference in activities of daily living, and frequency and change during past week.	8	5
Short-Form 36 Vitality [124]	Any adult population	A 4-item scale covering energy and fatigue.	1	1

#### Treatment

Given the multifactorial nature of RA-fatigue, interventions should be multifaceted and directed towards factors which may be exacerbating fatigue, such as pain or mood disturbance, in individual patients.

Although the relationship between disease activity and fatigue is complex, evidence suggests that biologic drugs do reduce fatigue. A systematic review by Almeida et al assessing the impact of biologic agents (20 TNF-inhibitors, and 12 non-TNF-inhibitors) on fatigue reported that biologics in patients with active RA can lead to small-to-moderate improvements in fatigue, with similar magnitudes of effect observed for both TNF-inhibitors and other biologic agents [90]. The authors concluded, however, that “it is unclear whether the improvement results from a direct action of the biologics on fatigue, or indirectly through reduction in inflammation, disease activity or some other mechanism”. More recently, similar modest effects on reducing fatigue have been reported with the Janus Kinase inhibitor, baricitinib [91].

A Cochrane systematic review of 24 studies examining non-pharmacological interventions for fatigue by Cramp et al found small but statistically significant benefits of both physical activity interventions and psychosocial interventions [92]. Another systematic review by Kelley et al of aerobic exercise as a treatment for RA-fatigue, suggested that whilst land-based aerobic exercise is associated with statistically significant reductions in fatigue, it is unlikely that large numbers of people would obtain clinically-relevant reductions [93]. They based their conclusion on changes in relation to the minimal important difference effect size and recommended cut-points. At the same time land-based aerobic exercise did not appear to increase fatigue and is safe; therefore, overall it is likely to be beneficial as part of the overall management of RA.

There is a resource implication in implementing many of these physical or cognitive behavioural approaches, which will limit their uptake within routine clinical care. A simple, more implementable approach to increasing exercise to target fatigue is the use of wearable-technology, such as pedometers. A clinical trial by Katz et al, suggested that this approach is effective in RA. In this trial, 96 patients were randomised to receive either education alone (control group), or a pedometer with step-monitoring diary, with or without step targets. Both intervention groups had significantly higher activity levels and greater reductions in fatigue at 21-weeks compared with the control group [94]. Overall the balance of evidence is strongly in favour of recommending RA patients exercise regularly to limit their fatigue. Although by itself it is unlikely to resolve this feature entirely, it is safe, effective and inexpensive and can be combined with other approaches.

### Muscle loss and RA Cachexia

#### Definition

There are two types of cachexia that can occur in patients with RA. The first is the “classic” low body mass index (BMI) form, in which patients with severe systemic disease lose both muscle mass and fat mass, leading to an emaciated appearance [95]. The second is “RA cachexia” in which muscle mass is low, but is compensated for by a gain in body fat.

#### Aetiology

Patients can lose muscle mass for several reasons, including malnutrition, starvation, cachexia and sarcopenia. Malnutrition and starvation are simple concepts related to insufficient food intake. Sarcopenia is predominantly age-related skeletal muscle loss, and is consequently often considered to be a geriatric syndrome [96]. In contrast, cachexia is the consequence of a long-term systemic inflammatory response. The key feature of cachexia is the redistribution of protein content, with skeletal muscle depleted of proteins and an increase in the synthesis of proteins related to the acute-phase response. RA cachexia is considered to be driven by the overproduction of cytokines and inflammation [97], with these metabolic changes of cachexia being cytokine-regulated [98]. RA cachexia has been linked to the metabolic syndrome, with associated abnormalities in lipid levels [99]. Patients with RA cachexia have abnormal energy and protein metabolism and increased inflammatory cytokine production including interleukin-1 and tumour necrosis factor [100].

#### Prevalence

In RA, there are marked variations in the reported prevalence of cachexia. Some experts suggest it is very common, occurring in as many as two-thirds of patients with RA [101]. Other experts have drawn different conclusions, and suggest it is relatively rare and only occurs in approximately 1% of patients [102]. It is likely that these differences are driven by the use of diverse criteria to define the presence of RA cachexia, with different studies using different definitions, based on varying fat and muscle mass cut-offs [99, 103]. Overall, however, classic cachexia is considered rare, and easily identifiable, and RA cachexia, is considered more common although it is not readily identified by patients and clinicians owing to the presence of a normal, or even increased, BMI [95].

#### Methods of assessment

Measuring weight and height provide useful information in many settings but are insufficient to assess muscle mass, which is needed to evaluate the presence of RA cachexia. Early studies used a variety of approaches to assess cachexia including energy expenditure profiles and whole body protein turnover [100]. The accurate assessment of cachexia in RA depends upon being able to define the amount of lean body mass, and fat mass that is present. Whole body imaging using computerised tomography and magnetic resonance imaging can achieve this goal but their use in large numbers of patients is impractical. Dual-energy X-ray absorptiometry, which is widely used to assess bone density in RA, is a reliable and established method for examining the composition of body soft tissue and determining how much is fat and how much is lean mass. It is, therefore, potentially valuable in larger clinical studies of RA cachexia, though it is not currently used for this evaluation in routine practice [104]. A simpler alternative is bioelectrical impedance analysis, which can accurately estimate body composition, particularly the amount of body fat. It determines the electrical impedance, or opposition to the flow of an electric current, through body tissues. This allows an assessment of the total body water, which can be used to estimate fat-free body mass and, by difference with body weight, the amount of body fat. It has been successfully employed in RA patients and is likely to be particularly useful in epidemiological studies [105].

#### Impact

The loss of lean body mass, a key component of RA cachexia, has been shown across several studies to strongly associate with the presence of disability. Engvall et al. reported that within 60 patients with RA, the correlation coefficient between lean body mass and HAQ scores was − 0.42 (*P* = 0.001) [103]. Other studies have also reported significant associations between loss of lean body mass and disability [106, 107]. The balance of evidence suggests that cachexia causes disability, but there are complex interactions between RA cachexia, sedentary lifestyles and disability in patients with RA. There is a growing body of evidence that sedentary behaviour, which means too much sitting as opposed to movement and exercise, may drive persisting inflammatory disease and elements of cachexia in RA [108].

RA cachexia is often considered to have detrimental impacts on cardiovascular health, although this issue appears controversial. Summers et al [95] have reviewed this relationship in detail, and they identified two studies reporting the association between RA cachexia and cardiovascular disease [99, 109]. The findings of these studies depended on the cut-offs of fat and muscle mass used to define rheumatoid cachexia. Taking a fat free mass index below the 25th percentile and fat mass index above the 50th percentile of a reference population, Elkan et al reported that within 80 patients with RA, 18% of women and 26% of men had “rheumatoid cachexia” and that these individuals had significantly higher total cholesterol and low density lipoprotein, alongside a higher frequency of hypertension and metabolic syndrome [99]. In contrast, using the same definition applied to 400 patients with RA, Metsios et al. reported no significant differences in cardiovascular risk factors, or established cardiovascular disease between patients with and without RA cachexia [109].

#### Treatment

As cytokines are implicated in the development of RA cachexia there has been considerable interest in evaluating whether cytokine inhibition can improve it. Two small studies evaluated this possibility. One represented a retrospective comparison of 20 RA cases receiving tumour necrosis factor inhibitors and 12 matched controls. Over 12 weeks, biologics improved disease activity and physical function but there were no significant changes in resting energy expenditure and fat-free body mass [110]. The other study was a small 6-month trial of etanercept in 26 patients with early RA; it provided no substantial evidence that this treatment had an important impact on cachexia, though there was some evidence that biologic treatment normalised the anabolic response to overfeeding in a minority of patients [111]. This finding implies that instead of excess food intake resulting in increases in body fat, lean body tissue is preferentially formed in these patients. A larger study of 82 patients subsequently evaluated the impact of tight control using treat-to-target approaches. It also found no evidence that this approach improved RA cachexia [112].The balance of evidence from these small studies is that inhibiting cytokines and controlling synovitis has little impact on RA cachexia, which requires an alternative management strategy.

The impact of exercise appears more positive. An initial small observational study of three months’ progressive resistance training as an adjunctive treatment for rheumatoid cachexia in 10 RA patients with matched controls showed it was effective and safe for stimulating muscle growth [113]. A subsequent trial of 28 patients with established, controlled disease showed six months’ weekly progressive resistance training was both safe and effective in restoring lean mass and function in these patients [114]. Follow-up of some of these patients at three years showed that stopping the resistance training and resuming normal activity resulted in loss of the benefits of progressive resistance training on lean mass and strength-related function. However, there was substantial retention of the benefits of reduced fat mass and walking ability [115]. Recent research has shown that a short six-week treatment using progressive resistance training can be readily achieved within routine care settings and that this approach is beneficial for patients [116]. The balance of current evidence favours this approach to treat RA cachexia.

### Assessing these extra-articular impacts in routine care

Pain, depression and anxiety, fatigue, and rheumatoid cachexia are important issues that would benefit from assessment and management in a routine clinic setting. Delivering this will be challenging, as there are already extensive time pressures in delivering the standard T2T approach. However, the growing use of electronic medical records, and digital technologies to capture PROs (reports of patients’ health that come directly from the patient and are measured using standardised, validated questionnaires [117]) that “feed forward” into these, may make this achievable within current medical resources. Although such PROs would not be able to directly identify patients with rheumatoid cachexia, they would identify patients with functional impairment likely to benefit from exercise therapy, which in turn would help improve any co-existing cachexia.

Such an approach, in a rheumatology context, has been pioneered by the Swedish Rheumatology Quality Registry [118]. Patients with rheumatic diseases (including RA) attending a number of clinics across Sweden are able to complete a self-administered health survey prior to their clinic review. This can be undertaken at their routine clinic review using a touch-screen computer in the waiting room area, or at home/work via a secure internet web portal. Patients enter data on a range of PROs, covering general well-being, pain, activities of daily living, quality of life, and ability to work. These patient reported data are then “fed-forward” into their electronic medical records, and summarised in a summary overview “dashboard”, which trends their PROs and clinician-reported outcomes over time. During their clinic appointment, the clinician and patient review the co-produced dashboard information together, decide on the next treatment steps, and print an updated summary overview for the patient to bring home. A questionnaire and qualitative interviews of a subset of patients and clinicians confirmed this system to be acceptable, and useful, with 96% of patients rating their “overall impression of the system” as “excellent” or “very good” [119]. A similar approach is being undertaken at the University of Manchester, using a mobile phone application (the Remote Monitoring of RA (REMORA) app), which allows patients to log daily symptoms of their RA and its impact between clinic appointments; these data are sent directly to their electronic healthcare records [120]. Positive feedback was gained from patients in preliminary testing, who felt that it made care “more personal to you”, and easier to have a “shared conversation” with the clinician [121]. Additionally, a high-level of data completeness was obtained over a 3-month period of testing [122]. Further research in this area is required, with key questions including which PROs should be measured in a routine NHS setting, how the information should be presented to patients and clinicians, and what management should be undertaken for identified problems.

## Conclusions

The evidence outlined in this review has demonstrated that pain, anxiety and depression, fatigue, and muscle loss, are highly prevalent problems in patients with RA. Whilst T2T has revolutionised the overall health and outcomes of patients with RA, it does not directly address these important extra-articular impacts, which can persist despite attaining remission. This suggests that these symptoms are likely to benefit from a more targeted management approach, which is used alongside T2T. This is in-line with patients’ preferences, with addressing pain being a key treatment goal across a broad range of patient surveys.

Research suggests that pain, mental health, and fatigue are inter-related problems, that share overlapping aetiologies. As such, they are likely to benefit from a holistic assessment strategy and treatment approach. As detailed in this review, there is evidence to support the use of non-pharmacological strategies, such as psychological interventions and specific forms of exercise to address these issues, with the latter also benefiting muscle loss. Although these interventions have, on the whole, small-to-modest clinical gains, if they are used in combination, and tailored to individual patients, their efficacy is likely to be optimised.

There are many challenges in delivering such a “holistic care” approach to patients. Key barriers include a lack of access to psychological services (with nearly three-quarters of rheumatology units in England self-rating their access to psychological support as being “inadequate”), time constraints in clinic (with follow-up appointments generally lasting 15 min), financial constraints within the NHS, alongside uncertainty as to who should be undertaking this (primary or secondary care clinicians).

Further research is required to clarify the optimal way to address these extra-articular impacts in routine care. This needs to not only focus on how to manage these issues, but also how they can be assessed within a brief clinic appointment. It is likely that digital technologies will play an important role in this area, enabling PRO data to be collected electronically and populated into patients’ electronic health care records. Although there is a risk of overwhelming clinicians with information in the short-term, clinical practice should rapidly adjust to incorporate this additional data. A focus on improving co-ordination of care across the primary-secondary care interface is also needed, to ensure that rheumatologists and community services with expertise in managing mental health, are working together in an optimal manner, for the good of patients.

## Abbreviations

BMI: body mass index
BP: bodily pain
BRAF MDQ: Bristol RA Fatigue Multi-Dimensional Questionnaire
BRAF NRS: Bristol RA Fatigue Numerical Rating Scales
CBT: cognitive behavioural therapy
DAS28: disease activity score on a 28-joint count
DMARD: disease-modifying anti-rheumatic drug
ESR: erythrocyte sedimentation rate
GAD: generalised anxiety disorder
GHQ-28: general health questionnaire
HADS: hospital anxiety and depression scale
HAQ: health assessment questionnaire
HR: hazards ratio
HRQoL: health-related quality of life
LDA: low disease activity
MDHAQ: multi-dimensional HAQ
MSK: musculoskeletal
MSK-HQ: musculoskeletal health questionnaire
NHS: national health service
NICE: national institute for health and care excellence
NRS: numeric rating scale
NSAIDs: non-steroidal anti-inflammatory drugs
PHQ-9: patient health questionnaire
PRO: patient reported outcome
PtGA: patient global assessment of disease activity
QOF: quality and outcomes framework
QUEST-RA: quantitative standard monitoring of patients with RA
RA: rheumatoid arthritis
RAPID: routine assessment of patient index data
RAPS: RA pain scale
REMORA: remote monitoring of RA
SF-36: short-form 36
SJC: swollen joint count
T2T: treat-to-target
TJC: tender joint count
VAS: visual analogue scale
VDS: verbal descriptive scale
